# Experimental Observations of Graphene at Phospholipid Monolayers

**DOI:** 10.1021/acs.langmuir.5c05780

**Published:** 2026-02-18

**Authors:** Amy D. Chacón, David M. Goggin, Joseph R. Samaniuk

**Affiliations:** Department of Chemical and Biological Engineering, 3557 Colorado School of Mines, Golden, Colorado 80401, United States

## Abstract

Interactions between particles and phospholipids at fluid–fluid interfaces play an important role in biological systems, nanotechnology, and consumer products. Two-dimensional (2D) particles are atomically thick particles whose interaction with phospholipid films is little understood and challenging to study experimentally. In this work we have quantified the diffusivity of graphene, a well-studied 2D particle, at a phospholipid monolayer using experimental methods. Particle diffusivities are used to identify how the graphene arranges in the phospholipid film. Diffusion coefficients of graphene particles were calculated from tracking their displacements at an air–water interface covered with Dipalmitoylphosphatidylcholine (DPPC) phospholipids. DPPC surface area concentration was varied, and diffusivities of the particles were compared with model predictions to deduce whether the particles were embedded within the phospholipid film or interacted primarily with one side of the film. Discrepancies between experimentally obtained diffusivities and theoretically predicted diffusivities suggest that the graphene particles fabricated in this work interact primarily with DPPC hydrocarbon tails rather than displacing DPPC molecules to embed within the film.

## Graphical abstract

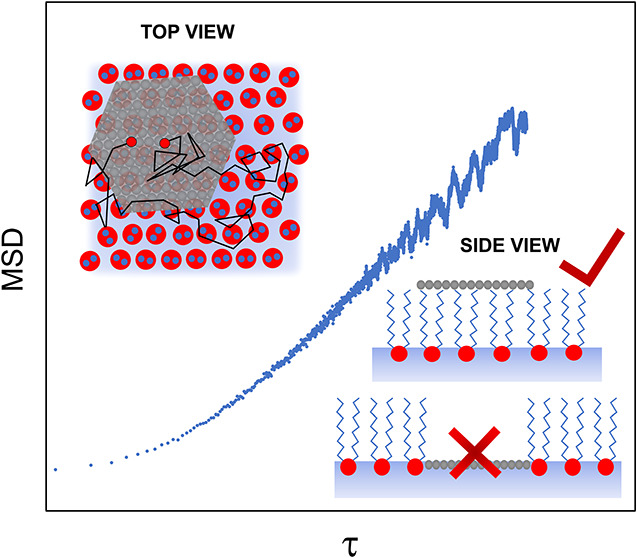

## Introduction

Interactions between particles and phospholipid films are important in materials science applications and in biological systems, but depending on the particle-phospholipid system, the nature of the interactions can be difficult to identify. Interactions may include fundamental forces such as Coulomb, and van der Waals, but they may also imply the way a particle arranges within a phospholipid film (e.g., interacting with the phospholipid hydrocarbon tails versus the polar headgroups or embedding or inserting in the film and displacing phospholipids). Knowing how a particle arranges with a phospholipid film is important for predicting the transport properties of the particle, which has important implications for understanding diffusion of proteins through biological membranes, the potential cytotoxicity of nanoparticles used as drug delivery vehicles, and the stabilizing effect of particles in Pickering emulsions. The diffusive behavior and arrangement of three-dimensional (3D) particles in thin films of phospholipids has been studied,^1^ but the focus of this work is on two-dimensional (2D) particles and their arrangement within phospholipid films, an area that is not well studied. Although there have been a number of computational investigations of 2D particle interactions with phospholipid membranes or thin films,^2–4^ there are few experimental works^5,6^ in part because of the lack of model experimental systems available and the challenge of obtaining microscopy video data on interfacially trapped 2D particles. In this work we fabricate a model experimental system to investigate the arrangement of particles of a well-studied 2D material, graphene, in a well-studied phospholipid monolayer of Dipalmitoylphosphatidylcholine (DPPC) at an air–water interface.

Two-dimensional (2D) materials are atomically thick crystalline materials that possess unique properties depending on their chemistry, structure, and thickness. The most commonly studied 2D material, graphene, has alterable surface chemistry, high tensile strength, a large surface area to volume ratio, optical transparency, and excellent electrical and heat conductance.^7,8^ Other examples include molybdenum disulfide (MoS_2_) and hexagonal boron nitride (h-BN), each with different properties. In general, 2D materials are appealing to use in applications such as sensors, electronics, energy, catalysis, and nanotechnology.^7–14^ Graphene and graphene-based materials are attractive for many reasons, including the ease with which they can be chemically functionalized, their ability to stabilize emulsions strongly and cost-efficiently in comparison to spherical particles^15–17^ , and their potential to be used as drug delivery vehicles.^5^ Graphene and other 2D materials are increasingly being used for composites, consumer products, and biosensors, making the interaction of 2D materials with biological systems more likely and more prevalent.^6,8,18,19^

As potential applications for graphene in consumer products and biomedicine grow, it is important to ask how graphene interacts with biological systems. Concern over unknowns and the lack of data with which to answer such question has led to temporary bans on face masks containing graphene, and though risk from inhalation of graphene is considered low, the risks are not well understood.^20^ Research with mice has shown that when graphene oxide is inhaled, it can lead to lung inflammation and oxidative stress.^14^ The reason for those biological responses is not completely understood. In addition to concerns over health impacts, understanding how 2D materials influence biological structures such as lipid monolayers and bilayers is important for potential applications like the use of 2D materials as drug delivery vehicles. Biological membranes are barriers by nature and utilizing particles of 2D materials for drug delivery requires that such a particle interact with cell membranes. Thus, understanding how 2D materials adsorb to biological membranes, how they diffuse along or through biological membranes, and what variables influence those physical interactions is critical. There are many open questions about how 2D materials impact human health, and how they behave when they encounter biological thin films, and answering those questions will be key for understanding the biological implications of their use.

Cell membranes are complex structures consisting of several biological molecules including lipids, proteins, and carbohydrates.^21^ Experimentally investigating the interaction of particles of 2D materials with cell membranes is challenging because of the complexity of the membrane structure and the challenge of visualizing the interaction of a subnanometer-thick particle in situ with the membrane. To facilitate experimental studies of biological membranes, many investigations have utilized a model membrane comprised of a single phospholipid such as DPPC.^22–28^ Such membranes can be formed as bilayers in thin film balances^29^ or as monolayers at air–water interfaces.^30^ DPPC is a common surfactant present in most lipid bilayer cell membranes, and in lipid monolayers in the lung alveoli. The presence of a phospholipid monolayer at the surface of the fluid films within alveoli helps to reduce surface tension and thus the force required to inhale. This monolayer consists of a mixture of different phospholipids and proteins, but is approximately 65 wt % DPPC.^31^ The preparation and study of DPPC monolayers at air–water interfaces is relatively simple, and such systems are well studied.^22–28^ DPPC monolayers are considered model systems for studying lung surfactant monolayers, and for experimentally investigating the way particles interact with biological membranes. In this work we are particularly interested in analyzing the nature of diffusion of 2D particles in DPPC monolayers because diffusion is related to the arrangement of the particle–membrane system and the associated drag on the particle. Additionally, various theoretical models exist that relate membrane and particle properties to particle diffusion, and such models can be tested with experimentally obtained diffusivities.

The diffusive behavior of 2D particles in membranes is related to the morphology of the particle–membrane assembly, and theoretical models developed for predicting the diffusive behavior of particles in films start with assumptions made about that morphology. Though many models exist that relate the diffusion of a particle in a membrane to membrane properties,^32,33^ the one most relevant to this work is the model by Hughes, Palithorpe, and White (HPW).^33^ This hydrodynamic model relates the diffusion of a cylindrical inclusion of arbitrary radius, *a*, that is embedded in a film of the same thickness, to the membrane or film interfacial viscosity, η, and the viscosities of the surrounding bulk fluids, μ_1_ and μ_2_. Although the HPW model may be used to predict 2D particle diffusivity at biological membranes, the calculations require computational methods since diffusivity is not made explicit in the model. Petrov and Schwille^34^ developed a highly accurate approximation to the HPW model that provides an explicit form for the particle diffusivity

1 $$D\left(\varepsilon \right)=\frac{\frac{K_{B}T}{4\pi \eta }\left(\ln\left(\frac{2}{\varepsilon }\right)-\gamma +\frac{4\varepsilon }{\pi }-\left(\frac{\varepsilon^{2}}{2}\right)\ln\left(\frac{2}{\varepsilon }\right)\right)}{\left(1-\left(\frac{\varepsilon^{3}}{\pi }\right)\ln\left(\frac{2}{\varepsilon }\right)+\frac{C_{1}\varepsilon^{b_{1}}}{\left(1+C_{2}\varepsilon^{b_{2}}\right)}\right)}$$

where *K*_B_ is the Boltzmann constant, *T* is temperature, ε is a ratio between the particle radius and the viscosities involved ε = *a*/(η/(μ_1_ + μ_2_)), γ is Euler’s constant, and *C*_1_, *b*_1_, *C*_2_, *b*_2_, are fitting parameters. For 2D particles in the shape of a disk, the Petrov and Schwille approximation should be a straightforward method for predicting the diffusive behavior of 2D particles embedded in a phospholipid membrane. Theoretical models are valuable for predicting particle diffusion in membranes, but they must be validated with experimental values. As will be discussed next, measurements of 2D particle diffusion in phospholipid films can be achieved with particle tracking methods.

To experimentally study the diffusion of 2D particles in phospholipid membranes, observation and tracking of particle movement can be done with microscopy. Colloidal particles experience a random motion when suspended in a fluid medium. For particles at equilibrium with their surroundings, diffusing freely in an isotropic environment, such motion is referred to as Brownian.^35,36^ In general, the random path taken by particles can be characterized with a Mean Square displacement (MSD)^37^

2 $$<\Delta r^{2}\left(\tau \right)>=2\mathrm{dD}\tau^{\alpha }$$

where Δ*r* is the displacement of the particle(s), τ is lag time, *d* is the number of dimensions in which the diffusion is occurring, *D* is particle diffusivity, and α is a scaling exponent.^38^ For Brownian diffusion, α = 1, for subdiffusion, α < 1, and for superdiffusion, α > 1. The MSD of particles can be experimentally measured and then related to *D* through eq 2. Computing the MSD of particles requires observation of the particles using microscopy and tracking their positions over time. Calculating particle diffusivity from experimentally measured MSD for 2D particles at phospholipid films can provide values with which to validate theoretical models like HPW and Petrov and Schwille. Disagreements between experimental results and theoretical predictions may be used to gain insight into the nature of the arrangement of particle and membrane, or to identify where certain assumptions that underpin the theory may be in question.

There are several recent studies that focus on understanding how graphene or graphene-oxide particles interact with phospholipid membranes. Merchán et al. utilized grazing incidence X-ray diffraction (GIXD), neuron reflectivity (NR), and X-ray reflectivity (XRR) to investigate the arrangement of 2D graphene oxide flakes in monolayers of DPPC and DPPC variants at air–water interfaces.^5^ They deduced the particle arrangement in the membrane by combining scattering techniques and theoretical models. Although scattering techniques can provide estimates of average locations of particles, there were no direct observations of the graphene oxide particles at the DPPC monolayers. They determined that altering properties of the monolayer, such as the area per molecule of the phospholipid, can be expected to influence the particle arrangement within or near a phospholipid film. It was observed that as the DPPC area per molecule decreased, graphene oxide preferentially interacted with the polar headgroups of DPPC and the water subphase rather than the hydrocarbon tails. They find that the intermolecular forces responsible for this interaction are both electrostatic attractive forces and dispersion forces. A study by Seth et al. was focused on determining the interactions between graphene oxide and reduced graphene oxide, and phospholipid monolayers of different charge for the purpose of biomedicine applications.^39^ Like Merchán et al., they used XRR and GIXD to determine the influence of these graphene-based materials on the structure of the membrane. They found that graphene oxide flakes interact with positively charged phospholipid membranes by strong electrostatics such that the particles lay horizontally and in proximity to the positively charged lipid headgroups, while reduced graphene oxide interacts favorably with a zwitterionic phospholipid membrane by inserting itself vertically into the region of the lipid tails. A key finding is that the arrangement of 2D particles in phospholipid membranes depends on the intermolecular interactions and the surface area concentration of the phospholipid.

Frost et al.^6^ studied the adsorption of graphene oxide flakes onto negatively and positively charged phospholipid bilayer membranes on silicon substrates. The authors used surface analytical techniques such as a quartz crystal microbalance with dissipation monitoring (QCM-D), dual polarization interferometry (DPI), and atomic force microscopy (AFM), to experimentally investigate these systems. Results showed that negatively charged graphene oxide flakes adsorb favorably to positively charged membranes by lying flat on top of the membrane, while no graphene oxide adsorption was observed on negatively charged membranes. Like Merchán et al. and Seth et al.,^5,39^ they attribute the particle–membrane interactions to electrostatics. The authors used the favorable interaction between graphene oxide and positively charged membranes to fabricate multilayered composite structures with potential biomedical applications.

Chen et al.^4^ used MD simulations to investigate the interaction of lipid bilayers with graphene and graphene oxide particles. Graphene sheets with lateral dimensions below, above, and at approximately the thickness of the bilayer membranes tended to insert into the cellular membrane without causing rupture of the membrane or global disruption of the phospholipids. The authors point out that strong dispersion forces between the nonpolar graphene and the nonpolar lipid tails of the membrane drives the system to maximize the interaction between carbon atoms on the graphene and the hydrocarbon tails, facilitating graphene insertion into the membrane. In the case of graphene oxide, the sheet can only partially insert into the membrane and preferred to localize near the membrane-water interface due to its more hydrophilic nature. When graphene oxide was placed near the membrane it led to membrane rupture. During rupture, phospholipids were expelled from the membrane and water molecules entered the membrane. The authors referred to the resulting hole in the membrane as a pore and found that graphene oxide particles could lead to the development of pores within membranes. In a similar study by Chen et al.^3^ computational simulations were performed to investigate the effect that oxidation of graphene oxide particles would have on their interaction with phospholipid bilayers. As the graphene oxide becomes more oxidized, it exhibited a transition in its diffusive behavior from Brownian motion at low levels of oxidation, to Lévy motion at intermediate levels of oxidation, to directional motion at high levels of oxidation. The authors showed with MD simulations that as the graphene oxide flake inserts itself more and more into the membrane, it forms pores, pushing the membrane phospholipids out of their original position, similar to what Chen et al.^4^ found. An important finding from both studies is that particle chemistry and size can influence how 2D particles will interact with a phospholipid membrane, and the presence of the particle can lead to membrane morphological changes such as pore formation that in turn influences the particle diffusion.

Current work investigating the interactions between graphene-based materials and phospholipid films varies in focus between understanding how particle size and chemistry, phospholipid and/or particle charge, and the presence of multiple membrane components, impact the particle arrangement in the membrane. While some experimental results in this field are published, computational work is more widely available.^2–4^ Most of the published work in this area is focused on phospholipid bilayers or liposomes, and very few works have considered phospholipid monolayers.^2–4,6^ The experimental work that is available generally does not rely on direct observation of individual 2D particles at biological membranes to determine the particle arrangement and/or interactions at the membrane. In addition, previous experimental and computational studies have focused on lateral particle sizes between a few nanometers and 5 μm.^5,6,40^ Currently there is very little experimental work investigating the interaction of graphene-based materials with phospholipid monolayers, and for all work including those that focus on phospholipid bilayers, there is little or no work done investigating lateral particle sizes beyond 5 μm. While many potential biological applications for 2D particles (e.g., targeted drug delivery) tend to be concerned with particles of nanometer scale, there are many applications involving 2D materials (e.g., composites, electronics, and sensors) where micron-scale or larger particles may be encountered and ultimately interact with biological systems.^7,8^ In addition, to fully understand the influence of 2D particle lateral size on membrane interaction, more experimental work with a range of 2D particle sizes is needed.

Comparing predictions of particle diffusivity from the Petrov & Schwille approximation with experimental data can help determine the arrangement of 2D particles at phospholipid thin films. The HPW model, and thus the Petrov & Schwille approximation, is based on the assumption that particles are cylinders embedded in the membrane with a no-slip condition. The result is that hydrodynamic resistance from the membrane acts fully on the particle as it diffuses in-plane with the membrane. If such a particle were not embedded in the membrane, but instead adsorbed to one side of the membrane, the assumptions underlying HPW would not necessarily be valid. In that case, both hydrodynamics and the influence of any slip condition at the particle-phospholipid contact surface would determine the drag on the particle. In this work we take the approach of comparing the predictions of Petrov & Schwille with data from a model experimental system to identify whether a micron-scale graphene particle will embed in a DPPC monolayer membrane or adsorb to one side of the membrane. The model experimental system is comprised of graphene monolayer disks fabricated with photolithography, and DPPC monolayers prepared from dropwise spreading at an air–water interface. Optical microscopy and particle tracking is used to measure diffusivity of the particles so that they can be compared with predictions from the Petrov & Schwille approximation. Ultimately, we find that monolayer graphene interacts primarily with the hydrocarbon tails of the DPPC monolayer rather than embedding in the membrane. The important consequence is that graphene particles in these systems diffuse orders of magnitude faster than the predictions from the most relevant theoretical models, necessitating the development of new models for these systems.

## Experimental Section

### Preparation of Air–Water Interfaces

Air–water interfaces were prepared in a custom sample chamber described in detail by Kale et al.^41^ The sample chamber had several components: a glass coverslip acting to hold the water subphase, a stainless steel insert with several steps to pin the air–water interface, and a top stainless steel ring to hold components together with screws. Prior to experiments, all parts of the chamber are thoroughly cleaned with solvents and ultrapure water with a resistivity of 18.2 MΩ·cm that was obtained with a PURELAB Ultra Analytical purification unit, ELGA LabWater, U.K. The glass coverslip was rinsed with reagent grade acetone, followed by reagent grade ethanol, followed by ultrapure water. This cleaning cycle was iterated twice. The stainless steel components were cleaned using the reagent grade ethanol followed by the ultrapure water, iterated twice. To prepare the air–water interface, a single-use 1 mL sanitary syringe was used to deposit ultrapure water up to the first step of the chamber.

### Spreading Polystyrene Microspheres at the Interface

Polystyrene microspheres were spread to help with identifying the location of the air–water interface. They were spread after the transfer of the graphene particles to the air–water interface, and prior to spreading DPPC. A 50/50 volumetric mixture of ultrapure water and isopropyl alcohol (ACS Reagent) was used to spread 1 μm diameter sulfate-functionalized polystyrene microspheres (Polybead, USA). The spreading solution was created by depositing 2–3 drops of Polybead’s stock solution into the 50/50 mixture. The solution was deposited carefully at the air–water interface without directly touching it using a 10 μL Hamilton syringe. 30 min were allowed to pass to allow for isopropanol evaporation. Some of the particles adsorbed to the air–water interface while others entered the water subphase.

### Particle Fabrication and Transfer to Air–Water Interface

To fabricate and transfer graphene particles of controlled shape and size to the air–water interface, the method developed by Goggin et al. was used.^42,43^ This method consisted of two parts: particle fabrication, and particle transfer to the air–water interface. Although the details of the multistep method can be found in the works mentioned, the specific settings for each step are included here. Prior to fabrication, a 8.5 mm by 8.5 mm piece of monolayer graphene grown via chemical vapor deposition on copper foil (Graphenea Inc., SanSebastiań, Spain) was dry etched with reactive ion etching (device model: Nordson March CS-1701) to remove graphene from one side, referred to as the backside. The conditions used were: a 40:10 SCCM Argon (Ar): Oxygen­(O_2_) gas mixture at a pressure of 0.2 Torr, with a power of 30 W, for a duration of 90 s. All additional processing steps were performed on the other side of the foil, referred to as the front side. The front side of the sample was coated with a layer of photoresist (AZ 1512, EMD Performance Materials, Integrated Micro Materials, Argyle, TX) approximately 1.2 μm thick with a spin coater (Cee Brewer Science 200CB Spin Coater). The spin recipe was as follows: (step 1) spin speed of 500 rpm, acceleration of 25 rpm/s, for 40 s, (step 2) 1000 rpm, 50 rpm/s, for 40 s, and (step 3) 4000 rpm, 200 rpm/s, for 80 s. The sample was then soft baked on a hot plate at 100 °C for 60 s to remove residual solvent and solidify the photoresist film. The sample was then taken to a mask aligner (SUSS MJB3, Karl Suss, Germany) for photolithography. The substrate was positioned directly below and in hard contact with a custom photomask (chrome photomask, with less than a 2 μm critical dimension, Front Range Photomask, Lake Havasu City, AZ) containing desired patterns (in this work, the sample was aligned to a pattern consisting of a 10 × 10 mm array of 10 μm disks). Disk shaped particles were chosen to minimize differences between the experiments and the theoretical models that assume cylindrical particles. The 10 μm particle diameter was chosen because the particles had measurable diffusivities using a standard microscopy setup over 10^0^ s time scales and it was within the limit of resolution of the photolithography patterning technique. A UV exposure dose of 125 mJ/cm^2^ was used. The sample was then immersed in developer solution (AZ 300 MIF, EMD Performance Materials, Integrated Micro Materials, Argyle, TX) for 45–60 s. The sample was cleaned in an ultrapure water bath for 5 min and dried with a flow of nitrogen gas. The front side of the foil was then dry etched to remove exposed graphene with the same method and conditions mentioned above. After etching, remaining photoresist was removed by immersing the sample in 1-Methyl-2-pyrrolidone (NMP) (Biotech. grade, purity is greater than or equal to 99.7%, Sigma-Aldrich) at 80 °C for 10 min. The resulting copper foil contained an array of 10 μm graphene monolayer disks. The foil was further cleaned by putting it in an acetone bath for 1 min, followed by an ethanol bath for 1 min, then by three separate ultrapure water baths for 5 min each. The sample was then left to dry at 21 °C.

Patterned graphene particles were transferred to an air–water interface using a wet etching process. The patterned copper foil substrate was placed at the surface of 0.25 M ammonium persulfate (APS) (ACS reagent grade, ≥98%, Sigma-Aldrich) at 40 °C for 2 h. The APS solution was prepared by dissolving solid form APS with 18.2 MΩ·cm ultrapure water. Prior to placing the foil substrate, a 2 mm thick fluoroelastomer (Viton) ring was placed floating on the surface of the APS. Then, the foil was placed floating within the ring. The ring had a 9 mm inner diameter and an 11 mm outer diameter. The ring provided containment of the foil and ultimately the graphene particles after the wet etching of the foil was complete. The band also facilitated transfer of the graphene particles to different subphases later. At least 3 h was allowed to pass so that APS could completely etch the solid copper foil, leaving only the patterned graphene particles at the air-APS interface. Transfer of the graphene particles was accomplished with a clean glass slide inserted into the interface underneath the fluoroelastomer ring at a shallow angle of approximately 30°. The glass slide was slowly lifted upward out of the interface, lifting the ring, the volume of APS inside of the ring, and the interfacially trapped graphene particles. The ring could be introduced to a new interface by gently submerging the glass slide at a shallow angle into the new subphase. In this way, the sample could be transferred to a series of clean air–water interfaces. For each transfer, the floating fluoroelastomer ring was gently moved around the new interface to encourage mixing of the bulk clean water with the small volume of bulk fluid transferred with the ring. The transfer process was repeated 3 times into 18.2 MΩ·cm clean water, before a final transfer to the sample chamber containing an air–water interface.

### Preparation of DPPC Monolayers

Monolayers of DPPC were prepared by dropwise addition of a bulk solution of racemic DPPC dissolved in chloroform. The bulk DPPC-chloroform solution was prepared by diluting a volume of 25 mg/mL stock solution (Avanti Polar Lipids Inc., USA) to 0.2 mg/mL with HPLC grade chloroform. One weight percent of Texas-red labeled 1,2-dihexadecanoyl-*sn*-glycero-3-phosphoethanolamine (DHPE) (Invitrogen, USA) was added to the solution to assist in fluorescence microscopy. A 5 μL Hamilton syringe was used to spread the solution dropwise to an initial concentration of 0.5 mg/m^2^ at the air–water interface within the fluoroelastomer band where the graphene disks were already located. The DPPC solution was always spread in small drops near the interface but without directly touching it. To increase surface concentration of DPPC, more solution was added dropwise. For each addition of DPPC, 30 min was allowed to pass to let any residual chloroform evaporate. The sequential addition process was repeated to achieve DPPC surface area concentrations from 0.5, 1.0, 1.5, 2.0, to 2.5 mg/m^2^. All experiments were conducted at a room temperature of 21 °C. Increasing the surface concentration of the DPPC monolayers by dropwise addition can cause disturbance to the fluid interface. We allow 30 min to pass after each addition to allow complete chloroform evaporation and for the system to equilibrate. It is unlikely that the phospholipids enter the bulk water subphase since DPPC molecules are highly insoluble in water.^44^ Also, the surface area concentrations of DPPC tested are not beyond monolayer collapse where multilayers and stable vesicle formation can be expected.^45^

Surface area concentration rather than surface pressure was relied on for several reasons. First, DPPC is highly insoluble in water, and thus a surface area concentration should be highly accurate. Second, surface pressure and surface area concentration in DPPC monolayers at air–water interfaces has been well characterized in past studies. Third, the use of a Wilhelmy probe, traditionally used to measure surface pressure at a fluid–fluid interface, causes a large meniscus to form that can disrupt the interface and make microscopy very difficult.

### Microscopy

Observation of fluid–fluid interfaces was done with both epifluorescence microscopy, and with interference reflection microscopy (IRM). The technique of IRM is described in detail in Goggin et al.^43^ The microscope used was a Nikon Ti–U Eclipse inverted (Nikon Inc., NY) with an ORCA-Flash 4.0 digital camera (C13440–20CU) (Hamamatsu Inc., NJ). A 10× objective with numerical aperture of 0.3 and a working distance of 16 mm (Nikon Plan Fluor, Nikon Inc., NY) were used for all experiments. NIS-Elements software (Nikon Inc., NY) was used for recording images and video. IRM required a custom microscopy filter cube described in Goggin et al.,^43^ and fluorescence microscopy of the Texas Red labeled DHPE required the Texas Red filter cube (Nikon Inc., NY). Switching between the two modes required simply rotating the filter cube turret to the appropriate location. Visualization of the graphene particles and the polystyrene particles was accomplished with IRM, while visualization of DPPC films required epifluorescence microscopy.

### Particle Tracking

A custom particle tracking code executed in MATLAB (MathWorks, Inc., MA) was used to calculate diffusivities of particles. Identification of particle centroids and creation of particle tracks was accomplished with MATLAB algorithms made available by Crocker and Grier.^46^ Tracking under the microscopy conditions used here provides approximately 10 nm precision for centroid identification.^46^ Mean square displacement (MSD), drift correction, image generation, and calculation of diffusivities was done with custom code. Images were obtained from videos, with each video taken at 83.3 frames per second (fps) for 10,0001 frames. The diffusivity in all experiments was calculated using MSD data near 1 s of lag time, as this avoided static noise at shorter lag times and the region of poor statistics at longer lag times. Static noise in captured images comes from variation in pixel intensities from frame to frame that results in an apparent movement of a particle centroid, even in the absence of particle movement. Static noise results in a measured value of MSD that is independent of lag time, and though it can vary with changes in the optics (e.g., illumination intensity, magnification), it can be quantified by immobilizing particles and imaging them with the optical settings that will be used for all measurements. If particle MSD approaches the same order of magnitude as the static noise MSD, then the static noise influences the measured MSD. At long lag times there are few observations of particle displacement, therefore the MSD values suffer from increasing variance. To obtain an approximate value of the static noise, a large surface area concentration in excess of 4 mg/m^2^ of DPPC was added to the interface, with images taken at the highest frame rates accessible. Although optical settings significantly influence the static noise limit, the minimum accessible values of MSD were approximately 0.01 μm^2^. For all measurements, four to seven graphene particles were tracked for 10,000 images for each DPPC concentration, and MSDs were time and ensemble averaged. Particle tracking was also done for the polystyrene spherical particles at 83.3 fps for 1000 images. Generally, 18 to 25 polystyrene particles were tracked for each DPPC concentration. Interfacial drift that occurs in experiments will result in a MSD that scales with lag time to a power greater than 1. An average-displacement subtraction was built into our in-house MATLAB tracking code to correct for global drift in the measurements. The average displacement of all tracked particles at each time step was computed and then subtracted from the individual particle displacements at each time step. In this way, the original particle track was modified to remove drift between each observation.

## Results and Discussion

IRM and fluorescence microscopy were used to quantify the diffusive behavior of 2D particles at DPPC monolayers at the air–water interface. IRM and fluorescence microscopy can be combined and used to visualize both the structure of DPPC monolayers and 2D particles present within the film. Figure 1 includes images (a–d) captured of an air–water interface with 10 μm diameter graphene disks and DPPC molecules at that interface. In image (a) IRM was used to resolve the 10 μm graphene disks and the 1 μm polystyrene spherical particles. Visualization of the DPPC monolayer structure was done with fluorescence microscopy as shown in image (b). In that image, bright red regions are liquid expanded (LE) domains of DPPC that are rich in Texas Red-labeled DHPE molecules, while dark regions are liquid condensed (LC) regions, rich in DPPC and few or no DHPE molecules.^22^ The LC and LE domains range from 1 to 5 μm in width, in agreement with other literature^23^ and with our previous work.^41^ Images (c) and (d) in Figure 1 are composite images where IRM and fluorescence images, taken only seconds apart, have been overlaid to better observe the local interactions between the DPPC monolayer and the graphene particles. Within those few seconds, the displacement of the graphene particles is between a tenth of a micron to a micron. Overlaying IRM and fluorescence images in this way can reveal how the graphene particles, the polystyrene particles, and the LE and LC domains partition and arrange with time.

**Figure 1:**
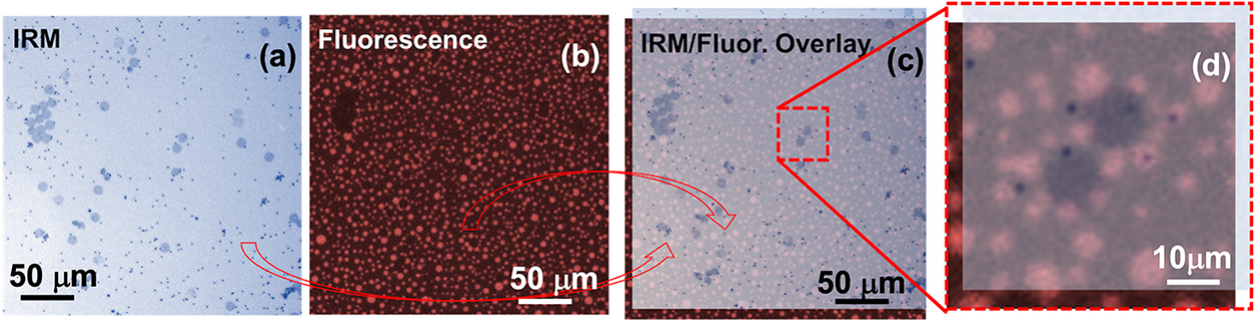
IRM and fluorescence microscopy images obtained for 10 μm graphene disks at an air–water interface with DPPC at a 0.5 mg/m^2^ surface concentration and 1 wt % Texas Red-DHPE. Image (a) was obtained with IRM and captures the location of the graphene particles and 1 μm polystyrene spherical particles. Image (b) is a fluorescence microscopy image showing LE and LC domains of the DPPC monolayer. Bright red domains seen in the image are LE domains, and dark domains are either LC regions or particles. IRM and fluorescence images obtained can be overlaid, shown in images (c) and (d) to discern between LC regions and particles, and to identify the local arrangement of the particles and the DPPC domains. Image (d) is a zoomed-in version of a segment of image (c) that shows two graphene particles, several polystyrene particles, and LE and LC regions of DPPC interacting.

Information on the way particles interact with a DPPC monolayer membrane can be obtained from analyzing microscopy images. For example, from Figure 1 it is apparent that the polystyrene particles do not preferentially interact with the LE domains, LC domains, or even the graphene particles. Some of the polystyrene particles appear to attach to the graphene disks, some aggregate with each other, and some diffuse individually. This was true for all DPPC surface area concentrations studied. Generally, the graphene disks were larger than the LE and LC domains, therefore, do not immerse themselves in either domain. In certain cases, when the LE domains or LC domains were larger than the characteristic size of the graphene particles, the particle would appear to be fully immersed in either domain. This was observed in the experiment with 1.0 mg/m^2^ DPPC (see Supporting Information Figure S1­(b)). Some computational results have shown that nanometer size graphene particles can preferentially associate with LE domains in DPPC:cholesterol mixed bilayers.^2^ One implication from the results here is that the characteristic size of the 2D particle and the phospholipid domains may play a role in this behavior. The images in Figure 1 reveal that both LE and LC domains in the DPPC monolayer associate with the edges of the graphene disks. Similar results are apparent at various monolayer surface concentrations (1.0, 1.5, 2.0, and 2.5 mg/m^2^) shown in Figure S1 of the Supporting Information. However, an important observation from the images in Figure 1 (also in Figure S1) is a lack of LE domains overlapping with graphene particles. This will be discussed more later, but the implication is that either the graphene particles displace phospholipids at the interface, or they prefer to associate with the densely packed hydrocarbon tails of the LC domains.

Microscopy allows for visualization of the location of particles and membrane features, but it also allows for quantification of particle diffusivities. Observations of particle positions over time can be used to calculate MSDs and diffusivities. Figure 2a is an IRM image of graphene disks and polystyrene particles at an air–water interface with a DPPC surface area concentration of 2.5 mg/m^2^. The tracks of four different graphene particles over a period of 120 s are shown in red. Figure 2b shows the track of particle 1 without any correction for global drift. The color mapping corresponds with the time of observation of the particle position, from 0 to 120 s. By comparing all four particle tracks in Figure 2a it is apparent that global drift of approximately 90 μm takes place over the 120 s of acquisition time, or less than 1 μm/s. Although drift could be mitigated, it needed to be corrected for in the data processing. Figure 2c shows the track of particle 1 with correction made for drift during data processing, as described in the Experimental Section. When global drift is corrected for, the particle track is Brownian in nature. The graphene particle tracks observed in all the experiments completed in this work were Brownian in nature when they were corrected for drift, with no evidence of directional motion or Lévy motion.

**Figure 2:**
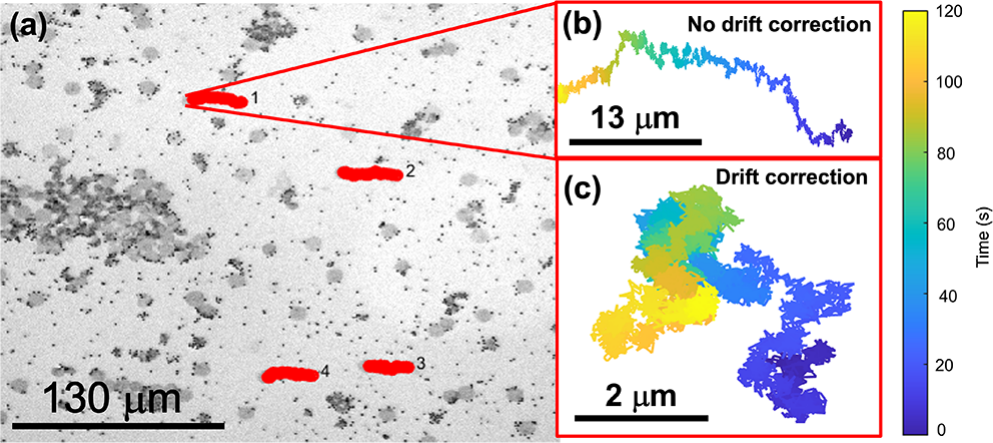
IRM microscopy utilized for tracking particles at DPPC monolayers. (a) An IRM image of four 10 μm graphene disk particles tracked at a DPPC thin film surface area concentration of 2.5 mg/m^2^ after 10,001 observations. The 1 μm polystyrene particles can also be seen. The four individual graphene particles were tracked without any drift correction (red lines) and are labeled 1 through 4 from top down. (b) The track for particle 1 without any correction for global drift. The color mapping corresponds with time from 0 s (blue, dark) to 120 s (yellow, light). (c) The track for particle 1 after correcting for global drift.

The drift-corrected tracks were used to calculate the ensemble and time averaged MSDs as a function of lag time and DPPC surface area concentration, as seen in Figure 3a. A general trend from the data was that MSD increases with lag time. At short lag times, below 1 s, the MSD values approach the static noise limit associated with the optical setup, thus the MSD appears subdiffusive. At longer lag times, above 10 s, the MSD becomes noisy, a result of limited statistics that occurs because of fewer observations of displacement at higher lag times. In between these two limits, the MSD depends on lag time linearly, with α ∼ 1. This confirms that the motion of the particles is Brownian in nature at these time scales. It is also apparent in Figure 3a that MSDs generally decreased with increasing DPPC surface area concentration from 0 mg/m^2^ (no DPPC) to 2.5 mg/m^2^. This trend is expected since increasing DPPC surface concentration increases the interfacial viscosity of the interface. The increased interfacial viscosity leads to increased drag on a particle and thus, slower diffusion. The drag on a graphene particle will depend on the arrangement of the particle in or around the phospholipid film. For example, a particle could be embedded in the DPPC film at the air–water interface, as is assumed by the HPW model and the Petrov and Schwille approximation, or it could be adjacent to the DPPC film where it associates with either the hydrocarbon tails or the polar head groups. Regardless of the arrangement, the general expectation agrees with the trend seen in Figure 3a that the particle will experience reduced diffusion as the surface concentration of DPPC increases.

**Figure 3:**
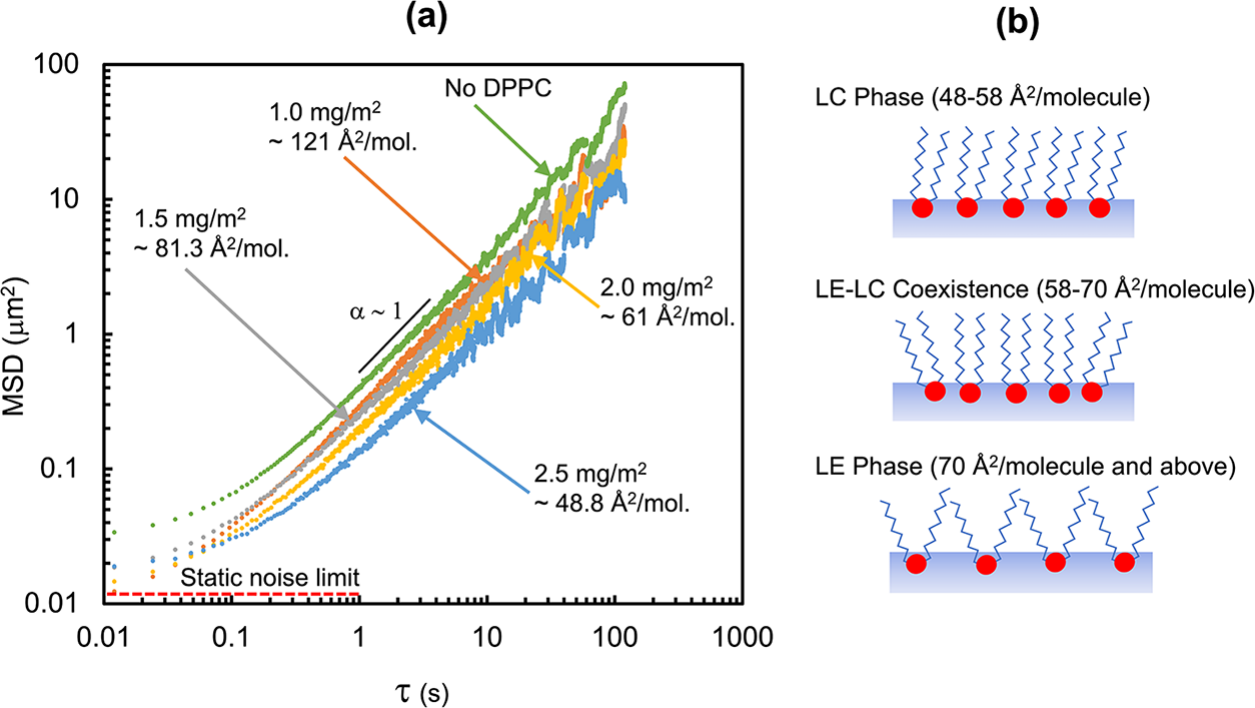
Mean square displacement (MSD) plots used to analyze the diffusive behavior of graphene particles at DPPC monolayers. (a) MSD plots for 10 μm graphene disks at DPPC monolayers as a function of DPPC surface area concentration. The curves show an overall linear relationship between MSD and lag time, with the exclusion of the static noise limit at short lag times and the statistically limited regions at long lag times. This figure implies that the 2D graphene particles display a Brownian diffusive behavior in the DPPC films. (b) Illustrations of the different arrangements of a DPPC film at the various areas per molecule where diffusion was observed with microscopy. At certain areas per molecule one expects various phases, including liquid expanded (LE), liquid condensed (LC), and LE-LC coexistence. A gas-like state of DPPC is expected to exist at areas per molecule greater than 90 Å^2^/molecule.

Although surface pressure was not measured in these experiments, the mean molecular area of DPPC can be calculated based on the mass of DPPC deposited on the interfacial area, enabling insight into the influence of membrane phase and particle diffusion. The mean molecular areas calculated from the mass of DPPC deposited, shown in Figure 3a, are robust since DPPC is insoluble in the aqueous subphase. Phases of DPPC at an air–water interface can vary from a gaseous phase, at greater than 90 Å^2^/molecule, to primarily a liquid condensed (LC) phase at areas per molecule between 48–58 Å^2^/molecule. Illustrations of the different arrangements of DPPC monolayers at these phases are provided in Figure 3b. MSD and thus, particle diffusivity, decreased with increasing DPPC surface area concentration, but no clear dependence of diffusivity on the DPPC phase was observed. We note that due to the complex nature of the particle fabrication and transfer, it is likely that the air–water interface was not perfectly clean prior to spreading of DPPC. Impurities can be encountered during the wet transfer process even after transferring to multiple clean water baths, including photoresist residues and large pieces of graphene where particles failed to form. Such impurities would effectively increase the actual DPPC surface area concentrations relative to the values reported here, which are based on the assumption of a clean air–water interface at spreading. Evidence for this can be seen in the microscopy images in Figure 1, where LE-LC coexistence regions are clearly observed at an apparent DPPC surface area concentration of 0.5 mg/m^2^ (>90 Å^2^/molecule), where a gas phase of DPPC would generally be expected. Although the actual DPPC surface area concentrations may be higher than the reported values, the main conclusions that follow were insensitive to that.

Mean square displacements of polystyrene particles were also tracked. Figure 4 includes MSD data for polystyrene particles and graphene particles at a DPPC surface area concentration of 2.5 mg/m^2^. There were also cases where polystyrene particles had visibly attached to graphene particles, and a MSD data set is also included in Figure 4 for such an aggregate at 2.5 mg/m^2^ DPPC. In addition to the MSD data, there are corresponding IRM images showing the different particles or aggregates tracked. Excluding the short lag time region influenced by static noise, and long lag times where the data is statistically limited, the MSD depends on lag time to a power of 1. This was true for all other particles and aggregates that were observed at other concentrations. Another observation is that the diffusivities of the graphene particles with and without attached polystyrene particles are very close, 0.034 ± 0.025 μm^2^/s (three particles tracked) and 0.035 ± 0.023 μm^2^/s (four particles tracked) respectively, and effectively the same. Diffusivities in both cases were calculated at a lag time of 1 s, and the plus/minus values are based on a single standard deviation of the ensemble and time averaged MSDs at that lag time. The MSD data of the individual polystyrene particles is greater than that of the individual graphene particles, including with or without polystyrene particles attached. At a lag time of 1 s, the diffusivity of the individual polystyrene particles is 0.15 μm^2^/s, a factor of 4 greater than the graphene particles.

**Figure 4:**
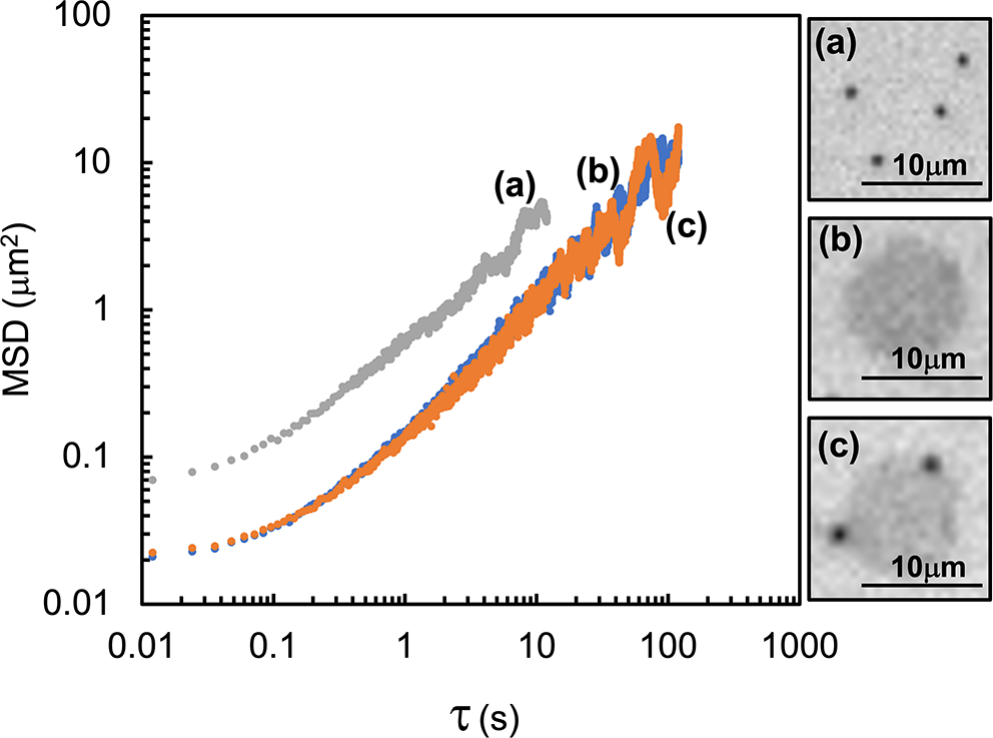
MSD as a function of lag time for (a) 18 individual 1 μm spherical polystyrene particles, (b) four individual 10 μm graphene disk particles, and (c) three graphene particles with two polystyrene particles visibly attached to the graphene particles that were tracked. DPPC was spread to a surface area concentration of 2.5 mg/m^2^. Corresponding IRM images reveal examples of some of the particles tracked for each data set.

The MSD’s and diffusivities of the polystyrene and graphene particles can provide evidence for how each arranges in the DPPC film. For polystyrene particles the diffusion appears independent of the DPPC surface concentration. Diffusion data for the polystyrene particles can be found in the Supporting Information, Figure S2. Previous work has shown that diffusivity of interfacially adsorbed polystyrene particles can be expected to decrease with increasing DPPC surface area concentration,^24^ but in that work DPPC surface area concentration was increased via compression of the interface from a lower surface area concentration, while in this work DPPC surface area concentration was increased with dropwise addition of DPPC from solvent. The dropwise addition of DPPC from a spreading solvent can result in significant disruption of the interface that can expel a previously adsorbed colloidal particle from an interface. The lack of decreasing MSD of the polystyrene particles with increasing DPPC concentration implies that the polystyrene particles likely were not adsorbed to the air–water interface and displacing DPPC molecules, instead diffusing along the hydrocarbon tails of the membrane.^24^ Since the MSDs of the graphene particles with and without attached polystyrene particles are essentially the same, and lower than that of the individual polystyrene particles, the implication is that the drag on the graphene particles is significantly greater than on the polystyrene particles, and that it dominates when the two different types of particles are aggregated. The trends observed in Figure 4 held true for all other DPPC surface area concentrations studied. It is important to note that the arrangement of particles at a membrane may be expected to vary depending on how the air–water interface is prepared experimentally.

Experimental diffusivity values of the graphene disk particles were compared with predictions from the Petrov and Schwille approximation. Figure 5 includes both experimental data from particle tracking, and theoretical predictions from the Petrov and Schwille approximation. Experimental diffusivities were calculated from MSD data in Figure 3, while the theoretical values were calculated from the Petrov and Schwille approximation. The DPPC interfacial viscosities utilized in the Petrov and Schwille approximation were obtained from Samaniuk and Vermant.^24^ In general, *D* decreases with increasing DPPC surface area concentration for both the experimental and theoretical values. An increase in the surface area concentration of DPPC should lead to an increase in interfacial viscosity and an increase in drag on particles interacting with the interface, thus lowering *D*. We also observe that experimental and theoretical *D* values do not agree above approximately 1.5 mg/m^2^. The graphene particles diffuse significantly faster than what the theory predicts. The sharp decrease in the predicted diffusivity is many orders of magnitude different between 1.5 mg/m^2^ and 2.5 mg/m^2^, but the decrease observed in experiments over that surface concentration range is only a factor of 2. A likely explanation for this discrepancy is that the graphene particles are not embedded in the DPPC layer, but are associating with one side of the DPPC monolayer membrane. A core assumption in the Petrov and Schwille approximation, and the HPW model that it is based on, is that the disk-like particle is embedded in the membrane. Consequently, it is subject to drag from the hydrodynamic contributions from both bulk phases and the membrane. If the disk-like particle instead associates with one side of the membrane such that it can slip at the surface of contact between particle and membrane, the HPW model would no longer be expected to apply, and the drag on the particle may be greatly reduced, and thus, the particle would diffuse faster. It is most likely that the graphene particles interact with the hydrocarbon tails of DPPC at these higher surface area concentrations. As mentioned earlier, in all fluorescence imaging (see Figures 1 and S1) there are no fluorescent domains (LE domains) that overlap with the graphene particles. It appears the particles preferentially overlap with the LC domains, a trend that holds at all monolayer surface concentrations. This can be expected given the chemistry of the system: van der Waals interactions dominate here, and the nonpolar graphene should be expected to maximize interaction with the tightly packed nonpolar hydrocarbon tails of the LC domains. This observation provides a chemistry-based argument in support of the hypothesis that the graphene particles arrange on the hydrocarbon-side of the monolayer. The inset illustrations in Figure 5 depict our suggested arrangement of the graphene particles diffusing on top of the lipid tails rather than embedding in the membrane as would be required for the HPW model. An important practical consequence of the finding here is that graphene particles diffuse at much higher rates along a DPPC monolayer membrane than current theory predicts. The ability to predict the diffusion of material through biological systems is important for bioengineering applications, thus the findings here have important implications for biological systems.

**Figure 5:**
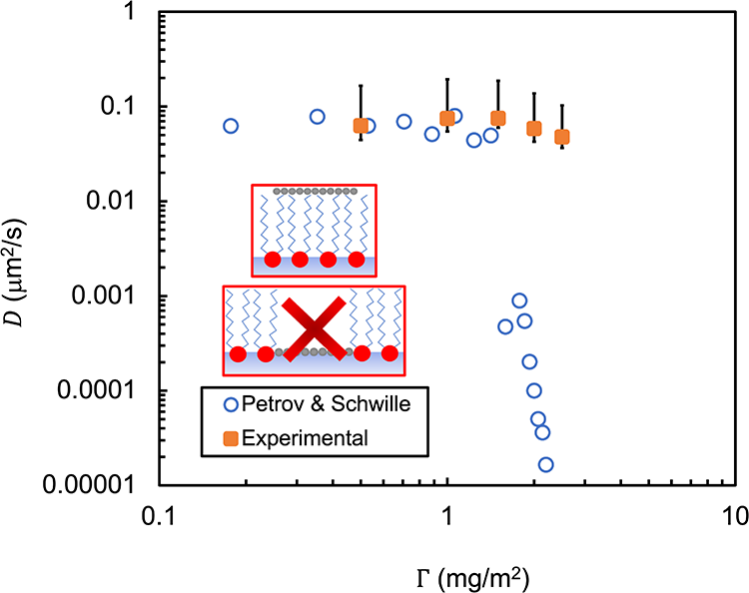
Particle Diffusivity, *D*, as a function of DPPC surface area concentration, Γ, for both experimental values and theory predictions of the Petrov and Schwille approximation. Experimental values of *D* (orange square symbols) are obtained from the MSD data in Figure 3 at lag times of approximately 1 s. The experimental diffusivity for 0 mg/m^2^ DPPC is 0.1 μm^2^/s. Error bars on the experimental data are calculated from a single standard deviation on the MSD value that was used to calculate the diffusivity for each DPPC concentration. Theoretical values of *D* (open blue symbols) are extracted from the Petrov and Schwille approximation using DPPC interfacial viscosities reported in Samaniuk and Vermant 2016. An approximately 10^3^ order of magnitude discrepancy is observed between experimental and theoretical values of *D* after a DPPC coverage of 1.5 mg/m^2^. As a result, the assumptions of the original HPW theory do not hold true for the 2D graphene particle-DPPC interaction system. The inset illustrations show our suggested arrangement of the graphene particles diffusing across the hydrocarbon tails of the DPPC monolayer rather than embedding in the monolayer like the HPW model, and thus, the Petrov and Schwille approximation, assumes.

Although unlikely, there are other possible contributions to the difference in diffusivities predicted from theory and measured experimentally. One possible contribution could come from the graphene particles localizing in either the LC or LE domains of the DPPC monolayer such that there would be a discrepancy between the microrheology and macrorheology of the system, that would in turn lead to the observations in Figure 5. While literature shows that the interfacial viscosities of the LC and LE domains are significantly different, this should not influence the conclusions reached in this work.^47^ The image analysis with fluorescence microscopy reveals that the graphene particles are not preferentially surrounded by either the LC or LE domains, and the particles are generally several times larger than individual LE and LC domains. Consequently, we can eliminate the influence of probe-localization in a domain as one of the reasons that the experimental diffusivities are much larger than theoretical diffusivities. Another possible explanation for the larger-than-expected diffusivities observed with the graphene particles could be local particle–membrane interactions such as the particle causing local yielding of the monolayer, but that is unlikely considering the simple chemistry and unfunctionalized nature of the graphene particle. An additional possible contribution could be an influence from particle thickness. Graphene particles are thinner than the DPPC monolayer, which violates an assumption in the HPW theory. Although the HPW model assumes the disk-like inclusion embedded in the membrane is the same thickness as the membrane, we assert that the thin nature of the graphene is unlikely to be the reason for the discrepancy between experiments and model. Compared to a membrane-thick inclusion, a thin inclusion (i.e., thinner than the surrounding membrane) would experience slightly different drag from either the superphase or the subphase, but the drag associated with translating in-plane through the surrounding flat membrane can be expected to be the same for thick or thin inclusions. Although it is unclear how large the differences in drag from the surrounding bulk phases would be, this would matter little at large interfacial viscosities where the drag of translating in-plane through the membrane exceeds the drag from the bulk phases by orders of magnitude. This ratio of drag from the interface and from the bulk phases is characterized with the Boussinesq number, and for monolayer DPPC membranes at surface concentrations beyond 1.5 mg/m^2^ the Boussinesq number is very large.^24^ In other words, we should expect a thin disk-like inclusion embedded in a thick, highly viscous membrane to experience very similar drag.

This work establishes the value of using experimental 2D particle diffusivity in conjunction with theoretical predictions to make inferences about the morphology of the system, but there remain several additional avenues of exploration beyond the scope of the current work. More work needs to be done to understand the influence of particle size and chemistry on the interaction of 2D particles and phospholipid membranes, as both can potentially alter where the particle arranges in the membrane. In addition, new diffusion models for 2D particles at interfaces that incorporate slip at the molecular level would be valuable in testing the current working hypothesis that micron-scale graphene particles diffuse along the hydrocarbon tails of a DPPC monolayer. Future work could also include an investigation of the influence of the order of operations for preparing the experimental system. The order in which particles and DPPC molecules are deposited could matter if a kinetic energy minimum exists that governs the position of the particles in or on the DPPC monolayer. Similarly, the method of changing the surface concentration of DPPC (i.e., dropwise addition versus mechanical compression of the monolayer from a dilute concentration of DPPC) could matter. The work here contributes an accessible experimental system and an appropriate data analysis scheme that can be used in future studies to address these open questions. Another area where more work needs to be done is in coarse graining MD simulations of systems with micron-scale 2D particles. Whole-atom MD simulations have been valuable in studying the interaction of nanoscale 2D particles with phospholipid membranes, but systems with micron-scale 2D particles are computationally too costly for whole-atom simulations.

## Conclusions

The diffusivity of graphene particles of controlled size and shape was experimentally measured in a DPPC monolayer at an air–water interface. This work is important because very little experimental data exists on the diffusivity of 2D particles in thin films of phospholipids, with no data available on model systems that can be compared with theoretical model predictions. The limited data is a consequence of the difficulty in fabricating a model experimental system to study, and in visualizing it. We fabricated 10 μm diameter graphene disks and transferred them to DPPC monolayers at an air–water interface. We used IRM and fluorescence microscopy to visualize and analyze the particle diffusion in the system. Using this model system, we characterized and quantified the particle diffusivity to understand the nature of these interactions, particularly how these particles arrange in the phospholipid. This was accomplished by measuring particle diffusivities and comparing values to predictions from the Petrov and Schwille approximation for particle diffusion in biological membranes. Comparing experimental and theoretical diffusivities, we found that the two differ by orders of magnitude depending on the surface area concentration, suggesting that the arrangement of graphene particles at a DPPC monolayer is different than what is assumed in the model. The results imply that graphene associates with the hydrocarbon tails of the DPPC monolayer, slipping along them rather than displacing the phospholipids and embedding in the monolayer. Our suggested mechanism was further supported by fluorescence microscopy images that revealed graphene preferentially overlaps with LC domains, as might be expected from the chemistry of the system. Ultimately our findings call into question the validity of the HPW model for this system, and they motivate the development of new theoretical models for 2D particle diffusion at biological membranes that can capture the effects of slip on the drag of the particles. This work contributes to the knowledge of the interaction of 2D materials with biological membranes, and in it we have identified several areas for future work including the influence of interface preparation, and the influence of 2D particle chemistry and size on the particle–membrane morphology.

## Supplementary Material



## Notes

Fluorescence microscopy images of the graphene-DPPC monolayer membrane model system at (a)­0.5-(e)­2.5 mg/m^2^ DPPC surface area concentrations (Pages S2–S3: Figure S1); mean square displacement (MSD) data of 1 μm polystyrene particles in the graphene-DPPC monolayer system at DPPC surface area concentrations between 0.5–2.5 mg/m^2^ (Page S4: Figure S2) (PDF)

## References

[1] Stone H. A., Masoud H. (2015). Mobility of membrane-trapped particles. J. Fluid Mech..

[2] Puigpelat E., Ignés-Mullol J., Sagués F. (2019). Interaction of Graphene
Nanoparticles and Lipid Membranes Displaying Different Liquid Orderings:
A Molecular Dynamics Study. Langmuir.

[3] Chen P., Yue H., Zhai X. (2019). Transport of a graphene nanosheet sandwiched
inside cell membranes. Sci. Adv..

[4] Chen J., Zhou G., Chen L. (2016). Interaction of Graphene
and its Oxide with Lipid Membrane: A Molecular Dynamics Simulation
Study. J. Phys. Chem. C.

[5] Dolores
Merchán M., Pawar N., Santamaria A. (2024). Structure of graphene oxide-phospholipid monolayers: A grazing incidence
X-ray diffraction and neutron and X-ray reflectivity study. J. Colloid Interface Sci..

[6] Frost R., Jönsson G. E., Chakarov D. (2012). Graphene Oxide and Lipid
Membranes: Interactions and Nanocomposite Structures. Nano Lett..

[7] Allen M. J., Tung V. C., Kaner R. B. (2010). Honeycomb
Carbon: A Review of Graphene. Chem. Rev..

[8] Akinwande D., Brennan C. J., Bunch J. S. (2017). A review on mechanics
and mechanical properties of 2D materials—Graphene and beyond. Extreme Mech. Lett..

[9] Stanford M. G., Rack P. D., Jariwala D. (2018). Emerging nanofabrication
and quantum
confinement techniques for 2D materials beyond graphene. npj 2D Mater. Appl..

[10] Bo T., Wang X., Jia R. (2021). Probing Activities of
Individual Catalytic Nanoflakes by Tunneling Mode of Scanning Electrochemical
Microscopy. J. Phys. Chem. C.

[11] Berweger S., Zhang H., Sahoo P. K. (2020). Spatially resolved persistent
photoconductivity in MoS2–WS2 lateral heterostructures. ACS Nano.

[12] Han X., Gerke C. S., Banerjee S. (2020). Strategic Design of
MoO2 Nanoparticles Supported by Carbon Nanowires for Enhanced Electrocatalytic
Nitrogen Reduction. ACS Energy Lett..

[13] Sharma M., Singh A., Singh R. (2020). Monolayer MoS2 Transferred on Arbitrary
Substrates for Potential Use in Flexible Electronics. ACS Appl. Nano Mater..

[14] Tabish T. A., Zhang S., Winyard P. G. (2018). Developing
the next generation of
graphene-based platforms for cancer therapeutics: The potential role
of reactive oxygen species. Redox Biol..

[15] Creighton M. A., Ohata Y., Miyawaki J. (2014). Two-Dimensional Materials
as Emulsion Stabilizers: Interfacial Thermodynamics and Molecular
Barrier Properties. Langmuir.

[16] Gonzalez
Ortiz D., Pochat-Bohatier C., Cambedouzou J. (2020). Current Trends in Pickering Emulsions: Particle Morphology and Applications. Engineering.

[17] Luo Q., Wang Y., Yoo E. (2018). Ionic Liquid-Containing
Pickering Emulsions Stabilized by Graphene Oxide-Based Surfactants. Langmuir.

[18] Xiao G., Li H., Yu Z. (2024). Highly thermoconductive, strong graphene-based
composite films by eliminating nanosheets wrinkles. Nano Micro Lett..

[19] Rathee M., Surendran H. K., Thakur A. (2025). Tailoring Functional
Graphene-Derived Geopolymer Nanocomposites: Interfacial Interactions
and Mechanical Strength Enhancement. ACS Mater.
Au.

[20] Estevan C., Vilanova E., Sogorb M. A. (2022). Case study:
risk associated to wearing
silver or graphene nanoparticle-coated facemasks for protection against
COVID-19. Arch. Toxicol..

[21] Watson H. (2015). Biological
membranes. Essays Biochem..

[22] Kim K., Choi S. Q., Zasadzinski J. A. (2011). Interfacial microrheology
of DPPC monolayers at the air–water interface. Soft Matter.

[23] Kim K., Choi S. Q., Zell Z. A. (2013). Effect of cholesterol
nanodomains on monolayer morphology and dynamics. Proc. Natl. Acad. Sci. U.S.A..

[24] Samaniuk J. R., Vermant J. (2014). Micro and macrorheology
at fluid-fluid interfaces. Soft Matter.

[25] Hermans E., Bhamla M. S., Kao P. (2015). Lung surfactants and
different contributions to thin film stability. Soft Matter.

[26] Choi S. Q., Steltenkamp S., Zasadzinski J. (2011). Active microrheology
and simultaneous visualization of sheared phospholipid monolayers. Nat. Commun..

[27] Casper C. B., Verreault D., Adams E. M. (2016). Surface Potential of
DPPC Monolayers on Concentrated Aqueous Salt Solutions. J. Phys. Chem. B.

[28] Shapovalov V. L. (1998). Interaction
of DPPC monolayer at air-water interface with hydrophobic ions. Thin Solid Films.

[29] Beltramo P. J., Van Hooghten R., Vermant J. (2016). Millimeter-area, free standing, phospholipid
bilayers. Soft Matter.

[30] Ciutara C. O., Iasella S. V., Huang B. (2023). Evolution of interfacial
mechanics of lung surfactant mimics progression of acute respiratory
distress syndrome. Proc. Natl. Acad. Sci. U.S.A..

[31] Griese M. (1999). Pulmonary
surfactant in health and human lung diseases: state of the art. Eur. Respir. J..

[32] Saffman P. G., Delbrück M. (1975). Brownian motion
in biological membranes. Proc. Natl. Acad. Sci.
U.S.A..

[33] Hughes B. D., Pailthorpe B. A., White L. R. (1981). The translational and rotational
drag on a cylinder moving in a membrane. J.
Fluid Mech..

[34] Petrov E. P., Schwille P. (2008). Translational diffusion in lipid membranes beyond the
Saffman-Delbrück approximation. Biophys.
J..

[35] Brown R. (1828). XXVII. A brief
account of microscopical observations made in the months of June,
July and August 1827, on the particles contained in the pollen of
plants; and on the general existence of active molecules in organic
and inorganic bodies. Philos. Mag..

[36] Piazza, R. Soft Matter: The Stuff that Dreams are Made of, 1st ed.; Copernicus: Dordrecht, 2011.

[37] Einstein A. (1906). On the theory
of the Brownian movement. Ann. Phys..

[38] Furst, E. M. ; Squires, T. M. Microrheology; Oxford University Press, 2017.

[39] Seth A., Mandal P., Hitaishi P. (2025). Assembly of graphene
oxide vs. reduced graphene oxide in a phospholipid monolayer at air–water
interfaces. Phys. Chem. Chem. Phys..

[40] Imperiali L., Liao K. H., Clasen C. (2012). Interfacial rheology
and structure of tiled graphene oxide sheets. Langmuir.

[41] Kale S. K., Cope A. J., Goggin D. M. (2021). A miniaturized radial
Langmuir trough for simultaneous dilatational deformation and interfacial
microscopy. J. Colloid Interface Sci..

[42] Goggin D. M., Samaniuk J. R. (2021). 2D Colloids: Size-
and Shape-Controlled 2D Materials
at Fluid–Fluid Interfaces. Langmuir.

[43] Goggin D. M., Zhang H., Miller E. M. (2020). Interference Provides
Clarity: Direct Observation of 2D Materials at Fluid–Fluid
Interfaces. ACS Nano.

[44] Wüstneck R., Wüstneck N., Grigoriev D. (1999). Stress relaxation behaviour
of dipalmitoyl phosphatidylcholine monolayers spread on the surface
of a pendant drop. Colloids Surf., B.

[45] Baoukina S., Monticelli L., Risselada H. J. (2008). The molecular mechanism
of lipid monolayer collapse. Proc. Natl. Acad.
Sci. U.S.A..

[46] Crocker J. C., Grier D. G. (1996). Methods of Digital Video Microscopy for Colloidal Studies. J. Colloid Interface Sci..

[47] Siebert T. A., Rugonyi S. (2008). Influence of Liquid-Layer
Thickness on Pulmonary Surfactant
Spreading and Collapse. Biophys. J..

