# Supplementary material for: Experimental Observations of Graphene at Phospholipid Monolayers
Source: Langmuir. 2026 Feb 18;42(8):6239–49. doi: 10.1021/acs.langmuir.5c05780 (PMC12961953; doi:10.1021/acs.langmuir.5c05780)
Supplement: Supplementary file 1 [file la5c05780_si_001.pdf]

SUPPORTING INFORMATION

Experimental observations of graphene at phospholipid monolayers

Amy D. Chacón, David M. Goggin, Joseph R. Samaniuk*

Department of Chemical and Biological Engineering, Colorado School of Mines, Golden, CO 80401, USA

*Corresponding Author: Joseph R. Samaniuk

Email: samaniuk@mines.edu

Table of Contents

- Page S2-S3: Figure S1 - Fluorescence microscopy images of the graphene-DPPC monolayer membrane model system at (a)0.5-(e)2.5 mg/m^2 DPPC surface area concentrations.

- Page S4: Figure S2 - Mean square displacement (MSD) data of 1 μm polystyrene particles in the graphene-DPPC monolayer system at DPPC surface area concentrations between 0.5-2.5 mg/m^2.

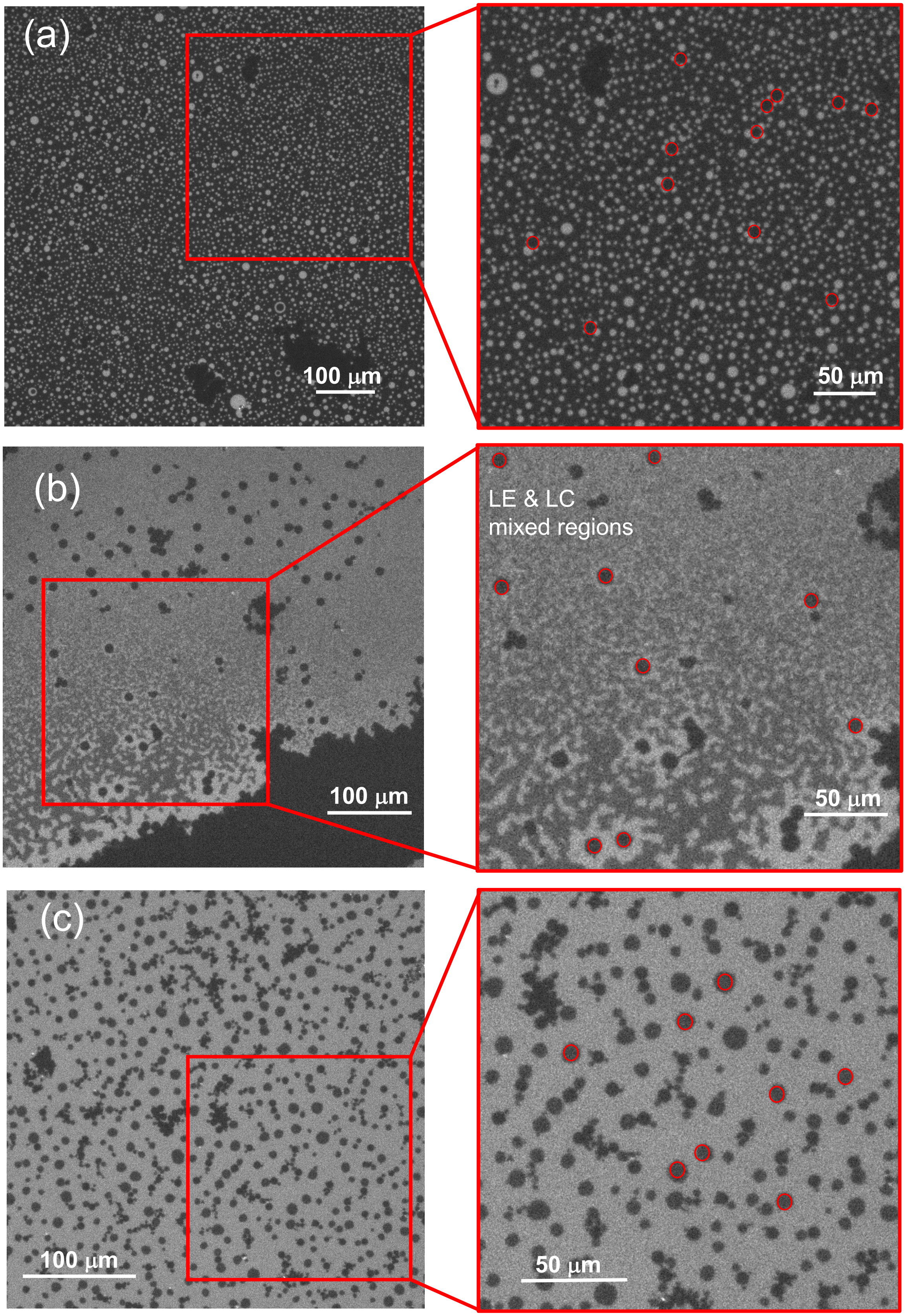
(a)
100 μm
50 μm
(b)
LE & LC
mixed regions
100 μm
50 μm
(c)
100 μm
50 μm

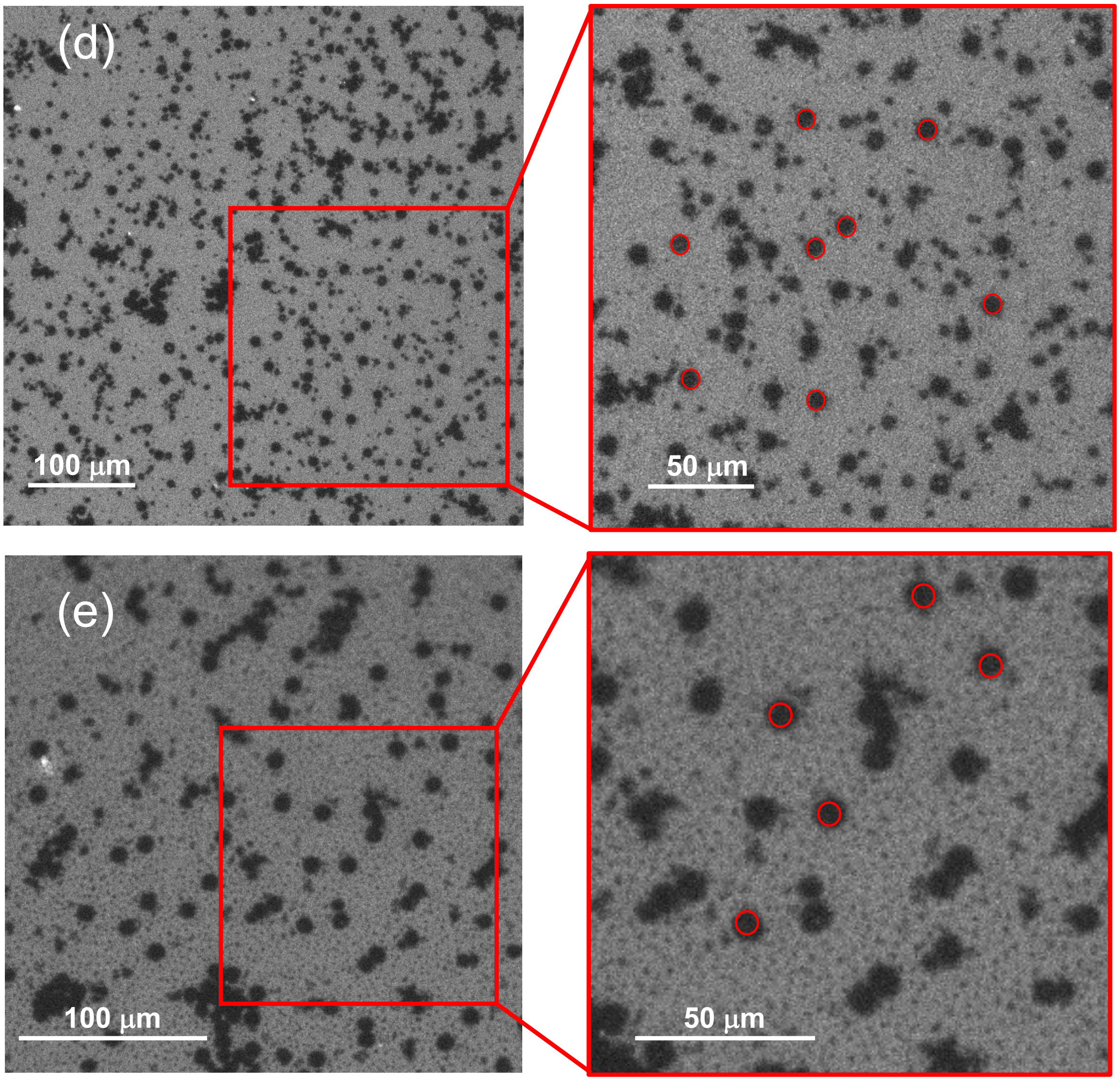

Figure S1: Fluorescence microscopy images captured for 10 μm graphene disks transferred to an air-water interface covered with DPPC at a surface area concentration of (a) 0.5 (b) 1.0 (c) 1.5 (d) 2.0 and (e) 2.5 mg/m^2. A zoomed in version of a segment of each image is provided as well as scale bars for each image. Darker regions in each image are either LC domains of DPPC, or particles, and lighter regions are the LE domains of DPPC that are rich in Texas red labeled DHPE. To differentiate between LC regions and particles, some of the graphene particles that were tracked are indicated with red circles. Generally, the graphene particles are larger than the DPPC domains and can be found associating with both domains without an apparent preference. When the graphene disks are smaller than either the LC or LE domains, the particles can arrange within the domains, as is apparent in the image at 1.0 mg/m^2 DPPC.

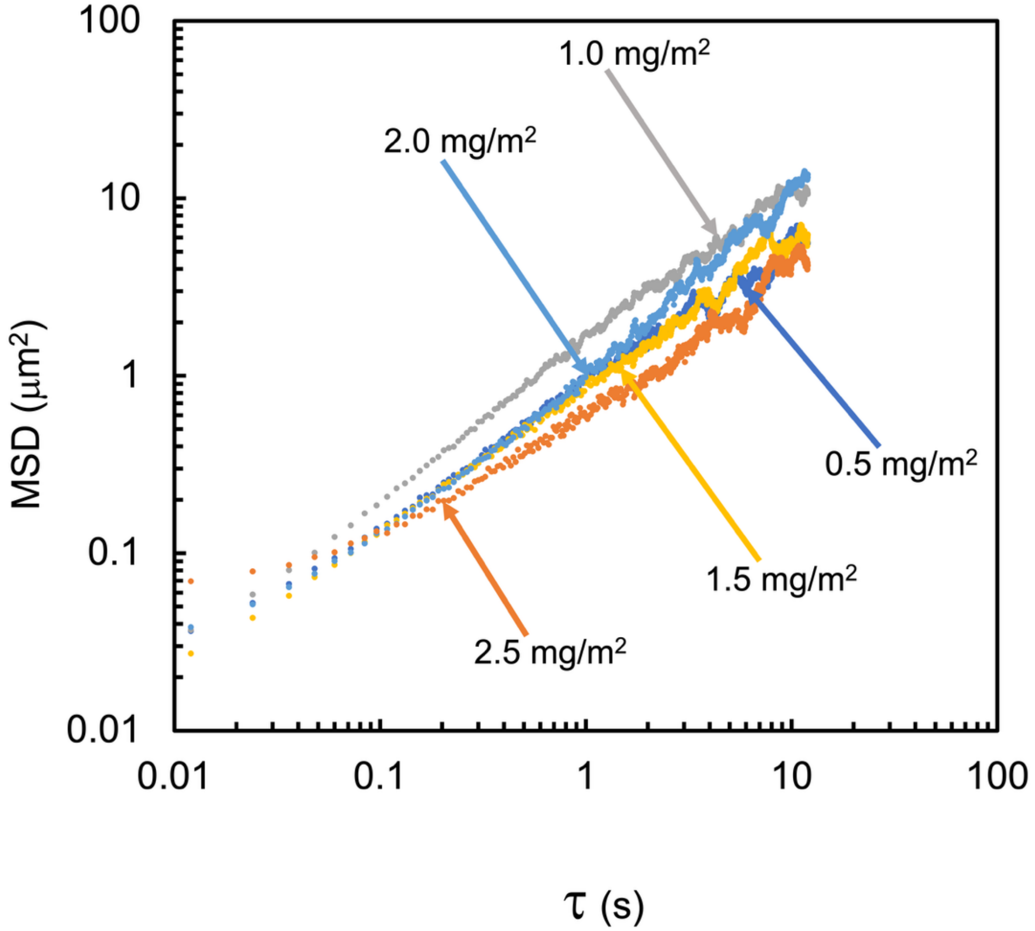

Figure S2: Mean square displacement (MSD) plots for 1 μm polystyrene microspheres at DPPC monolayers as a function of DPPC surface area concentration. In general, there is a linear relationship between MSD and lag time, with the exclusion of the static noise limit and the statistically limited regions. This implies that the particles display a Brownian diffusive behavior in the DPPC films. This figure also shows that there is not an evident trend between the MSD plots and DPPC surface area concentration.
